# Biological hydropersulfides and related polysulfides – a new concept and perspective in redox biology

**DOI:** 10.1002/1873-3468.13090

**Published:** 2018-05-24

**Authors:** Jon M. Fukuto, Louis J. Ignarro, Peter Nagy, David A. Wink, Christopher G. Kevil, Martin Feelisch, Miriam M. Cortese‐Krott, Christopher L. Bianco, Yoshito Kumagai, Adrian J. Hobbs, Joseph Lin, Tomoaki Ida, Takaaki Akaike

**Affiliations:** ^1^ Department of Chemistry Sonoma State University Rohnert Park CA USA; ^2^ Department of Molecular and Medical Pharmacology Center for the Health Sciences UCLA School of Medicine Los Angeles CA USA; ^3^ Department of Molecular Immunology and Toxicology National Institute of Oncology Budapest Hungary; ^4^ Tumor Biology Section Radiation Biology Branch National Cancer Institute Bethesda MD USA; ^5^ Department of Pathology Louisiana Statue University Health Sciences Center Shreveport LA USA; ^6^ NIHR Southampton Biomedical Research Center University Hospital Southampton NHS Foundation Trust Southampton UK; ^7^ Cardiovascular Research Laboratory Department of Cardiology, Pneumology and Angiology Medical Faculty Heinrich Heine University Dusseldorf Germany; ^8^ Environmental Biology Section Faculty of Medicine University of Tsukuba Ibaraki Japan; ^9^ William Harvey Research Institute Bart & London School of Medicine Queen Mary University of London Charterhouse Square London UK; ^10^ Department of Biology Sonoma State University Rohnert Park CA USA; ^11^ Department of Environmental Medicine and Molecular Toxicology Tohoku University Graduate School of Medicine Sendai Japan

**Keywords:** cysteine persulfides, hydropersulfides, translational polysulfidation

## Abstract

The chemical biology of thiols (RSH, e.g., cysteine and cysteine‐containing proteins/peptides) has been a topic of extreme interest for many decades due to their reported roles in protein structure/folding, redox signaling, metal ligation, cellular protection, and enzymology. While many of the studies on thiol/sulfur biochemistry have focused on thiols, relatively ignored have been hydropersulfides (RSSH) and higher order polysulfur species (RSS_n_H, RSS_n_R, *n* > 1). Recent and provocative work has alluded to the prevalence and likely physiological importance of RSSH and related RSS_n_H. RSSH of cysteine (Cys‐SSH) has been found to be prevalent in mammalian systems along with Cys‐SSH‐containing proteins. The RSSH functionality has not been examined to the extent of other biologically relevant sulfur derivatives (e.g., sulfenic acids, disulfides, etc.), whose roles in cell signaling are strongly indicated. The recent finding of Cys‐SSH biosynthesis and translational incorporation into proteins is an unequivocal indication of its fundamental importance and necessitates a more profound look into the physiology of RSSH. In this Review, we discuss the currently reported chemical biology of RSSH (and related species) as a prelude to discussing their possible physiological roles.

## Abbreviations

**3MST,** 3‐mercaptopyruvate sulfurtransferase

**CARS,** cysteinyl tRNA synthetases

**CBS,** cystathionine beta‐synthase

^**−**^ **CN,** cyanide ion

**CSE,** cystathionine gamma‐lyase

**GSH,** glutathione

**GSSH,** glutathione hydropersulfide

**GSSSG,** glutathione trisulfide

**GSSSSG,** glutathione tetrasulfide

**MST,** mercaptopyruvate sulfur transferase

**SeCys,** selenocysteine

The chemical biology of thiols (RSH, e.g., cysteine and cysteine‐containing proteins/peptides) has been a topic of extreme interest for many decades due to their reported roles in protein structure/folding, redox signaling, metal ligation, cellular protection, and enzymology 1, 2, 3, 4, 5. That is, oxidation of thiols to covalently linked disulfides (RSSR) is a critical aspect of protein folding and structure, modification (typically oxidative) of critical thiols can result in alteration of protein function/activity, thiol sulfur atoms are used in the binding of metals in numerous metalloproteins, thiol peptides scavenge otherwise toxic electrophiles, and thiolates (RS^−^) serve as potent nucleophiles important in the catalytic activity of numerous enzymes. Moreover, oxidized thiol species, such as thiyl radicals (RS·), sulfenic acids (RSOH), and sulfinic acids (RS(O)OH) also possess important chemical properties that Nature has exploited for various purposes. For example, RS· is used as an oxidant in the biosynthesis of DNA 6 and RSOH/RS(O)OH bind specific metals in metalloproteins, conferring activity 7. Thus, the diverse chemistry of thiols and derived species are fundamental to various aspects of cell biochemistry, function, and signaling.

In the recent past, most studies of thiol/sulfur biochemistry have focused primarily on RSH/RS^−^, RS·, RSSR, RSOH, nitrosothiols (RSNO), RS(O)OH, and sulfonic acids (RS(O)_2_OH). Relatively ignored have been hydropersulfides (RSSH) and higher order polysulfur species (i.e., RSS_n_H, RSS_n_R, *n* > 1, note: the term “hydropersulfide” and the abbreviation “RSSH” will be used to denote both the protonated and deprotonated species unless otherwise specified). Indeed, until very recently, the discussion of the chemical biology of hydropersulfides and polysulfides has been primarily relegated to studies of alliums and other food sources rich in these species 8 and to scant and specific examples of proteins with presumed hydropersulfides (of unknown function) 9, 10, 11. Also, hydropersulfides have been implicated in the biochemistry of several specific enzymes, such as mercaptopyruvate sulfur transferase (MST) 12, NIFS (a bacterial enzyme involved in metallocluster biosynthesis 13) and rhodanese 14, among others 15, some of which with no established physiological function. In spite of these examples, the idea that hydropersulfides are fundamental signaling/biochemical intermediates with important physiological functions (akin to previously mentioned sulfur species, such as sulfenic acids, disulfides, or S‐nitrosothiols) has not been generally considered. To be sure, important papers by Toohey, Wood and others, some of them published more than 25 years ago 16, 17, 18, indicated the possible general importance of hydropersulfides in biological systems. In spite of these somewhat prophetic reports, the idea that hydropersulfides (and related/derived polysulfides) are important and general biological mediators/effectors has remained unappreciated. However, there has been a resurgence of this idea due to recent and substantive reports of their biological prevalence and likely importance. In particular, a new and watershed report demonstrates translational cysteine hydropersulfide incorporation into proteins (*vide infra*), consistent with the idea of fundamentally important physiological roles for hydropersulfides. This finding, along with other recent work on the unique chemical and biological properties of hydropersulfides and related polysulfides, serves as the impetus for providing a perspective in redox biology.

It is the tenet of this article that hydropersulfides and related polysulfides represent a greatly underappreciated and fundamentally important aspect of sulfur‐based biochemistry/signaling that is involved in a diverse array of physiological and/or biochemical functions. Moreover, future elucidation of these effects and functions will lead to a more profound and complete understanding of the general utility of sulfur chemistry in biological/mammalian systems. Herein are discussed some of the recent reports indicating that hydropersulfides and polysulfides can play a role in mammalian biology, as well as the possible future directions of this field.

## The generation and prevalence of hydropersulfides and polysulfides in eukaryotes

The past and apparent lack of general interest in the biology of per‐ and polysulfides likely stemmed from the fact that their natural existence was only indirectly alluded to (based on nonspecific assays) and limited to only a select number of enzymes/proteins such as xanthine oxidase 10 and aldehyde oxidase 11. Until recently, the ubiquitous and highly prevalent nature of polysulfur species in mammalian systems was unknown. Recent and significant interest in the biological actions and signaling of hydrogen sulfide (H_2_S) 19 spurred interest in H_2_S‐related and/or derived species as possible biological mediators/effectors. One of the most important early studies regarding H_2_S pharmacology and physiology was by Blackstone and coworkers 20 who reported that pharmacological administration of H_2_S induced a reversible hibernative state in mice. This study revealed that low levels of H_2_S can have dramatic physiological effects and is not just a toxic species (which is observed at higher levels). An important aspect of the presence of H_2_S in biological systems is the possibility that RSSH and higher order polysulfides can be formed 21, 22.

One of the earliest and seminal studies drawing attention to the possible prevalence of polysulfur species (specifically hydropersulfides) in proteins was by Mustafa and coworkers 23 who utilized a modified biotin‐switch assay to detect protein hydropersulfides and suggested that 10–25% of numerous liver proteins contained protein hydropersulfides (this assay, as conducted, may not have been specific for hydropersulfides 2, 24). These investigators also reported that protein “sulfhydration”^1^ led to enhanced activity for glyceraldehyde 3‐phosphate dehydrogenase (although brought into question 25, 26) and promoted actin polymerization. Subsequent reports generally support the idea of the ubiquitous and highly prevalent nature of hydropersulfides and polysulfides in biological (i.e., eukaryotic) systems. For example, Ida and coworkers 27 utilized electrophilic trapping followed by LC‐MS analysis to find that small molecule hydropersulfides such as cysteine hydropersulfide (Cys‐SSH) and glutathione hydropersulfide (GSSH) as well as higher order (additional sulfur atoms, e.g., RSSSH) hydropersulfides were present in mammalian cells, tissue, and plasma. Indeed, levels of GSSH were found to be greater than 50 micromolar in mouse heart and liver and greater than 150 micromolar in mouse brain. Levels of Cys‐SSH, although significantly lower, were found to be in the low micromolar range in mouse heart, liver, and brain. Using a tag‐switch‐tag method 27, Ida and coworkers also detected numerous protein hydropersulfides, confirming the results of Mustafa and coworkers that the existence of protein hydropersulfide is widespread. Assuming the value of GSSH levels in mouse brain to be accurate (i.e., > 150 micromolar), GSSH may be the most prevalent cysteine‐containing sulfur species behind only glutathione (GSH) itself (which is typically between 1 and 10 mm in cells) in certain tissues.

The Nagy laboratory also developed a hydropersulfide‐specific assay, referred to as the protein hydropersulfide detection protocol (ProPerDP method), amenable to whole cells and tissues and confirmed the existence and prevalence of protein hydropersulfides in cells at levels in a steady‐state range of approximately 1–12 micrograms/milligram protein 28. An advantage of this method compared to others developed previously is that it utilized an alkylation step prior to cellular lysis, thus precluding cell lysis‐mediated artifactual oxidation/persulfidation. Further work by Longen and coworkers 29, also using a mass spectrometry‐based assay (entitled quantitative PerSulfide Site Identification or qPerS‐SID), found that cells treated with H_2_S resulted in the generation of numerous protein hydropersulfides (with significant identification overlap with the above‐mentioned work by Ida and coworkers 27). They also found that H_2_S‐mediated protein hydropersulfide formation appeared to exhibit specificity among numerous potential “sulfhydration” sites. That is, among numerous thiols on a single protein, only certain thiols were subject to hydropersulfide formation. Thus, there appears to be little doubt of the biological prevalence of hydropersulfide formation in peptides and proteins. This contention is supported by the finding by the Akaike group 30 of translational incorporation of cysteine hydropersulfide into proteins. Indeed, it is this major discovery that provides much of the impetus for writing this perspective, which will hopefully draw attention to this provocative discovery and its relevance to the field of sulfur biochemistry and physiology.

Up until 2017, hydropersulfide formation in peptides and proteins was thought to result primarily from a post‐translational modification of existing cysteine residues in proteins. That is, reaction of an oxidized protein cysteine (possibly a disulfide or sulfenic acid, among others) could react with H_2_S giving the corresponding hydropersulfide (Reactions 1 and 2).

(1) $$\text{R‐S‐S‐R}+H_{2}S⇌\text{RSSH}+\text{RSH}$$

(2) $$\text{RSOH}+H_{2}S\to \text{RSSH}+H_{2}O$$

These reactions have been studied extensively 21, 22, 31, 32 and they represent possible routes of endogenous hydropersulfide formation. It needs to be mentioned, however, that formation of hydropersulfides *via* Reaction 1 has been questioned on the basis of the low reducing power of H_2_S, its relatively low concentration and the high concentrations of GSH 33. Regardless, since H_2_S is biosynthesized *via* a variety of enzymes (*vide infra*) and the presence of oxidized sulfurs (i.e., disulfides, sulfenic acids) in biological systems is well established, generation of hydropersulfides *via* Reactions 1 or 2 is clearly possible. Indeed, hydropersulfides are likely involved in numerous equilibria with other sulfur species and, although at low steady‐state levels, are biologically accessible *via* these equilibria 34. However, it remains to be determined whether hydropersulfide formation *via* these reactions (or others) is significant and serves a biological function. That is, does Nature generate hydropersulfides as a response or reaction to some physiological state, cellular stress, or biochemical requirement? Do hydropersulfides serve a biological function and are, therefore, generated in a regulated manner?

**Figure 1:**
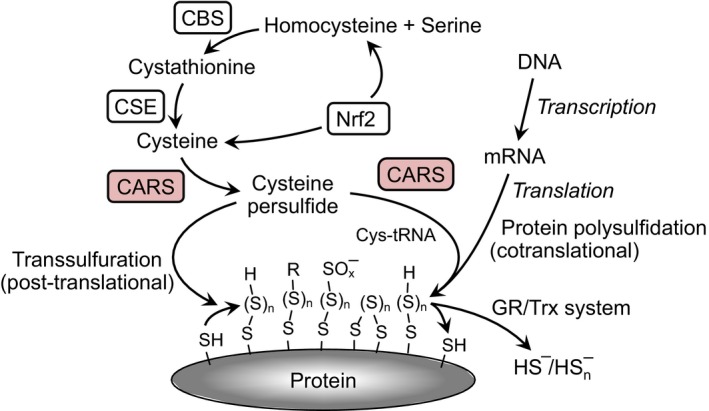
A schematic drawing for the cotranslational formation of Cys‐SSH and protein polysulfidation as catalyzed by CARSs. Cysteine persulfide (Cys‐SSH) formation and protein polysulfidation are mediated by the novel Cys‐SSH‐producing enzyme CARS. CBS and CSE are involved in the cysteine biogenesis in canonical transsulfuration pathway. Cystine transport by cystine‐glutamate transporter system mediated by xCT is also sustaining the intracellular cysteine. Cysteine biosynthesis and cystine uptake are known to be up‐regulated by Nrf2 (NF‐E2‐related factor 2), and the persulfides and polysulfides formed are reductively metabolized by glutathione reductase/thioredoxin (GR/Trx) system.

**Figure 2:**
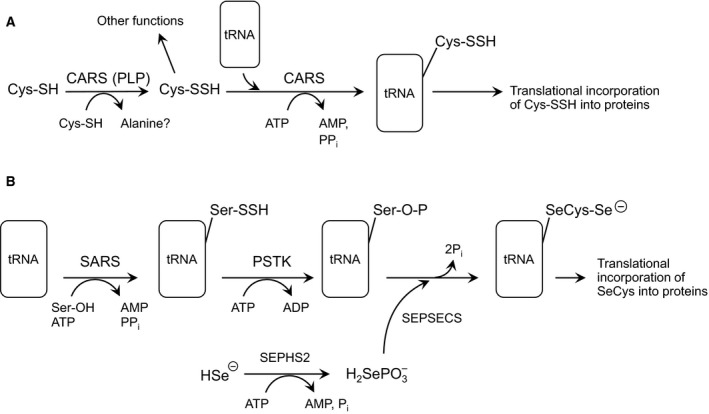
Schematics of the biosynthetic pathways for A) Cys‐SSH and B) SeCys. Cysteine tRNA synthetase (CARS); Serine tRNA synthetase (SARS); Phosphoseryl‐tRNA kinase (PSTK); Selenophosphate synthetase 2 (SEPHS2); Sep (O‐phosphoserine) tRNA; SeCys (Selenocysteine) tRNA synthetase (SEPSECS).

These questions have recently been answered by the finding that protein hydropersulfides are incorporated during translation *via* the actions of cysteinyl tRNA synthetases (CARS) 30 (Fig. 1). The Akaike group has provided strong evidence that CARS are capable of converting Cys‐SH to Cys‐SSH *via* a pyridoxal phosphate‐dependent process using a second Cys‐SH as the sulfur atom donor (independent of ATP and tRNA). The CARS‐synthesized Cys‐SSH can then form a tRNA‐bound Cys‐SSH adduct (also *via* CARS catalysis) resulting in the incorporation of Cys‐SSH into proteins, thus generating a protein containing a hydropersulfide functionality. This process is reminiscent of, although not mechanistically analogous to, the incorporation of selenocysteine (SeCys) into selenoproteins 35, 36. Figure 2 schematically illustrates and compares the mechanisms of Cys‐SSH and SeCys generation *via* translational machinery. A fundamental difference between the incorporation of Cys‐SSH and SeCys into proteins is that SeCys is biosynthesized from a tRNA‐bound serine while Cys‐SSH is premade from Cys‐SH by CARS prior to association with the tRNA, which is also catalyzed by CARS. Regardless, both SeCys and Cys‐SSH are biosynthesized by translational “machinery” prior to incorporation into proteins. The fact that hydropersulfides are both biosynthesized and attached to tRNA by CARS and subsequently translationally incorporated into proteins indicates they have a potential biological action and are not merely an artifact of H_2_S biosynthesis.

Considering the analogy between SeCys and Cys‐SSH, it is worthwhile to further compare these two amino acids. SeCys is considered to be Nature's 21st amino acid since it is biosynthesized pretranslationally (i.e., is not a post‐translational modification), specifically encoded (using UGA, which is normally the stop codon) and incorporated into proteins to serve a specific function. Analogously, Cys‐SSH may represent Nature's 22nd amino acid (in eukaryotes) for most of the same reasons (pyrrolysine is considered to be the 22nd amino acid in Archaea 37). However, the case for Cys‐SSH as the 22nd amino acid in eukaryotes is not yet as strong as it is for SeCys since the nature of the codon, the regulation of the process and the mechanism(s) that allows for specific incorporation are not yet established. Interestingly, based on their chemical properties, SeCys and Cys‐SSH can be considered to be functionally similar. Comparing these amino acids to Cys‐SH, they are both superior nucleophiles, more potent reductants, and more acidic (i.e., exist primarily in the anionic form). In the case of SeCys, all of these properties have been reported to be important with respect to its biological functions 38. For example, the high nucleophilicity and reducing capabilities of SeCys are critical to the activities of the redox proteins glutathione peroxidase and thioredoxin reductase. The same would be expected for the biological utility of protein hydropersulfides. However, an important distinction between SeCys and Cys‐SSH is the fact that Cys‐SSH can be easily converted to Cys‐SH, thus generating a “normal” cysteine protein (i.e., Reaction 3 or Reactions 4 and 5), whereas SeCys is essentially a permanent functional group on selenoproteins. Thus, Cys‐SSH can be viewed as a potentially fleeting SeCys‐like functionality allowing proteins containing hydropersulfides to change their chemical properties depending on, for example, the cellular redox environment.

(3) Protein−SSH+RSH→Protein−SH+RSSH

(4) $$\text{Protein}-\text{SSH}+\text{RSH}\to \text{Protein}-\text{SS}-R+H_{2}S$$

(5) Protein−SS−R+RSH→Protein−SH+RSSR

Previous to the discovery of Cys‐SSH formation *via* CARS, the biosynthesis of Cys‐SSH was known to occur primarily *via* the actions of two enzymes, cystathionine beta‐synthase (CBS) and cystathionine gamma‐lyase (CSE). These enzymes appear to be responsible for at least a portion of the Cys‐SSH found in cells since, for example, CSE knock out cells were significantly lower in intracellular hydropersulfides (and polysulfides) and CSE overexpressing cells possessed high levels 27. Previous work by Sen and coworkers using an improved maleimide‐based assay also showed that CSE‐delete mice lacked the ability to form hydropersulfides on the p65 subunit of NF‐κβ 39, affecting activity and Mustafa *et al*. 23 also reported that CSE expression led to hydropersulfide formation in proteins such as GAPDH, β‐tubulin, and actin. The idea that CSE and/or CBS are generally responsible for the Cys‐SSH found in cells has been questioned since the levels of enzymatic substrates and the reported kinetics of the enzymes would predict that H_2_S is the major product and not Cys‐SSH. That is, both CSE and CBS are somewhat promiscuous enzymes capable of catalyzing numerous reactions and based on the kinetic constants for these reactions and the approximate steady‐state levels of all the possible enzymatic substrates and reactive species, it has been argued that Cys‐SSH is merely a fleeting intermediate. It should be noted, however, that this treatment may oversimplify the system and does not, for example, account for other factors, such as Cys‐SS‐Cys uptake and redox regulation of the enzymes. Of significant importance, however, is the recent finding by Akaike and coworkers who have also demonstrated that CARS‐mediated Cys‐SSH biosynthesis can be responsible for much of the intracellular Cys‐SSH 30. In fact, this group has proposed that, in some cases, biosynthesis of Cys‐SSH by CARS may be responsible for the majority of the Cys‐SSH found in cells and tissues *in vivo*. Regardless of how hydropersulfides are made, it is clear they are prevalent (*vide supra*) and likely to possess important biological function.

Thus far, the discussion has focused almost exclusively on RSSH species. It is also noteworthy that polysulfides (RSS_n_R’, *n* > 1, R’ = hydrogen or alkyl) are prevalent in biological systems. Indeed, Ida and coworkers 27 have found numerous small molecule polysulfides (e.g., glutathione trisulfide (GSSSG), cysteine trisulfide (aka thiocystine, Cys‐SSS‐Cys), glutathione tetrasulfide (GSSSSG), cysteine tetrasulfide (Cys‐SSSS‐Cys), etc.) in human and mouse cells, tissue, or plasma. Moreover, the kinetic analysis of the enzymes CSE and CBS mentioned above also predicts that polysulfides are likely products. The mechanism(s) for the generation of these species in biological systems has not been determined and is likely to be due to a complex series of reactions involving, initially, hydropersulfides and possibly inorganic polysulfide species (e.g., ${\text{HS}}_{2}^{-}/S_{2}^{2-},{\text{HS}}_{3}^{-}/S_{3}^{2-}$, etc.).

## The chemical biology of hydropersulfides and polysulfides

**Figure 3:**
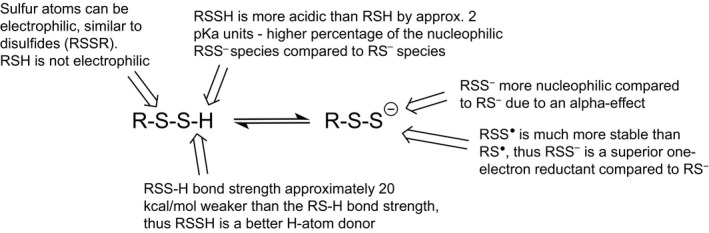
Comparative properties of RSS^−^/RSSH versus RS^−^/RSH.

The biological actions of hydropersulfides and polysulfides will undoubtedly be due to their unique and distinct chemistry. Thus, in order to speculate as to the biological functions of hydropersulfides and polysulfides, it is essential to first have a discussion regarding the chemistry of hydropersulfides and polysulfides and compare these chemical properties to other biologically relevant sulfur species. As mentioned above, hydropersulfides are superior to thiols as nucleophiles and reducing agents (Fig. 3). Although not yet established, it can also be predicted that the metal ligation properties are distinct. It has been proposed that RSSH is equivalent to a chemically hyperactivated RSH which led to the idea that “anything a thiol can do, a hydropersulfide can do better” 25. Although this can be why hydropersulfides are made in some cases (to enhance the activity of thiol proteins), it is at least safe to say that hydropersulfides possess distinct chemistry from that of simple thiols and that this may be important to the biological utility of hydropersulfides. One important distinction between thiols and hydropersulfides is the fact that thiols can be nucleophilic while hydropersulfides can be either nucleophilic or electrophilic depending on the protonation state. That is, a persulfide anion (RSS^−^) is extremely nucleophilic while the protonated RSSH species is electrophilic (akin to an oxidized sulfur species like a disulfide or sulfenic acid). Thus, hydropersulfides have the properties of both thiols and disulfides. It is also noteworthy that RSSH is oxidized relative to RSH (RSSH is at the same oxidation state as RSSR) and yet RSSH/RSS^−^ is a superior reductant compared to RSH/RS^−^. It may seem counterintuitive that the oxidation of a molecule results in the generation of a superior reductant, but this is exactly the case with the conversion of a thiol to a hydropersulfide. Indeed, this situation led to the idea that RSSH generation can be a biological response to cellular oxidative stress since an oxidized species generated under this stress is actually a superior reductant capable of possibly protecting against potentially deleterious oxidation events (due to its reducing capabilities, *vide infra*).

Thiyl radicals (RS·) are used biologically as potent oxidants capable of, for example, breaking a C‐H bond *via* hydrogen atom abstraction 6 and thus initiating radical chemistry. It has been postulated that RS· species may be involved in fundamental cellular redox signaling as well 40. The fact that RS· is a fairly potent one‐electron oxidant is an indication of the relatively poor ability of RSH to serve as a reductant. On the other hand, perthiyl radicals (RSS·) are relatively weak oxidants and are readily formed from one‐electron oxidation of RSSH/RSS^−^ 41, 42. RSSH/RSS^−^ is a good reductant due to the stability of the oxidized RSS· species. The stability of RSS· is akin to the stabilization of analogous nitroxide systems (R_2_NO·). In both cases, the stability of the unpaired electron is likely due to an overlap of the orbital containing the unpaired electron with the adjacent orbital containing a lone pair of electrons 43. The lack of reactivity of RSS· is evidenced by the fact that it does not readily react with O_2_ and NO, both established scavengers of other radical species 41, 44. Although it has been proposed that the one‐electron redox properties of RSSH/RSS^−^ may have biological function (i.e., can serve as a “redox gate” 41), this has yet to be shown.

**Figure 4:**
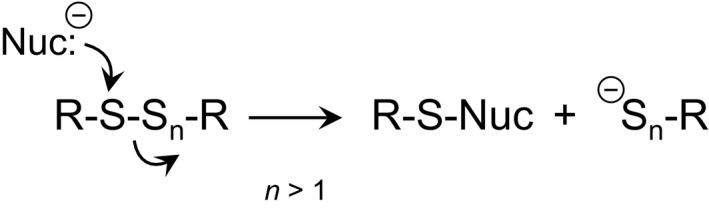
Reaction of a nucleophile with a polysulfur species.

Thus far, only the chemical biology of hydropersulfides has been discussed. As briefly mentioned above, polysulfides are also prevalent (albeit at lower levels than, for example, Cys‐SSH or GSSH). Like disulfides, polysulfides are electrophilic and thus readily react with nucleophiles. As “soft” electrophiles, these sulfur‐based species react primarily with “soft” nucleophiles, such as thiols, phosphines and cyanide 45, 46. The reaction of a nucleophile with a polysulfide presumably occurs *via* direct displacement of a sulfur containing leaving group (Fig. 4). For example, considering the reaction of an electrophilic disulfide with an appropriate nucleophile (e.g., a thiolate), the site of nucleophilic attack is primarily governed by the quality of the corresponding leaving group. That is, the most electrophilic sulfur, which is the site of nucleophilic attack, is the one that is attached to the best leaving group (i.e., the thiolate with a conjugate acid having the lowest pK_a_) 47. In the case of higher order polysulfides, the same trend is expected. Importantly, the pK_a_ of an RSSH species is typically 1–2 pK_a_ units lower than the corresponding RSH species 42 (and possibly much lower 32), indicating that reactions with an RSS^−^ leaving group should predominate over a reaction with an RS^−^ leaving group. Thus, an RSS_n_R (*n* > 1) species should be more electrophilic than the corresponding disulfide. It is also important to note that increasing the length of the polysulfur chain should also increase the inherent nucleophilicity of the sulfur atoms (due to an extended alpha‐effect 19, 48). Thus, an RSSSR species should be more nucleophilic compared to a simple disulfide. Although this is chemically expected and justifiable, quantitation of this phenomenon has not been reported. Regardless, the chemical biology of higher order polysulfides can be distinct from disulfides and these differences may be important to their biology (although this has not been specifically addressed thus far).

## Physiological implications and future work

Hydropersulfides are biosynthesized, incorporated into proteins and present as small molecules/peptides. Numerous descriptive studies have appeared in the literature reporting that the presence of hydropersulfides in proteins can elicit a functional change. Despite this, the overriding question remains: What is it that hydropersulfides specifically do? What is unique about hydropersulfides that prompted Nature to adopt them and evolve numerous synthetic processes to generate them (both as small molecule species and in proteins)? Is there something unique and novel about the hydropersulfide functional group that distinguishes it from, for example, thiols and/or selenols and are these unique properties a necessary aspect of fundamental biological/physiological signaling? At present, there are no clear or definitive answers to these questions, although some speculative proposals have been forwarded based on what is currently known regarding the distinct and novel chemistry of these species (*vide infra*). For example, the enhanced reductive capabilities and nucleophilicity of hydropersulfides compared to thiols may predict that their formation could be the result of cellular exposure to oxidative and/or electrophilic stress. Interestingly, proteins involved in Cys‐SSH biosynthesis can be induced under oxidative/electrophilic stress, consistent with the idea that their synthesis can be a response to these stresses 34. In this regard, it is important to reiterate that a hydropersulfide is an oxidized thiol and yet is a superior reductant compared to a thiol. That is, the oxidation of a thiol to a hydropersulfide, which could occur under oxidizing cellular conditions, generates a superior reductant. The seemingly incongruous and counterintuitive situation that a superior reductant is made *via* oxidation may be an important factor to the biological utility of hydropersulfides. To be sure, this is not a completely novel chemical phenomenon. For example, oxidation of an amine can generate a hydroxylamine, which is a superior one‐electron reductant compared to the corresponding amine. The generation of a hydropersulfide in the face of oxidative/electrophilic stress may serve to counteract the deleterious effects associated with these stresses, akin to the way glutathione/glutathione transferase scavenges electrophiles, and antioxidants such as ascorbate or the tocopherols limit oxidative radical damage. Importantly, Paul and Snyder proposed that hydropersulfide formation in proteins may possibly protect the protein cysteine thiol from oxidation under oxidative stress 49. However, the chemical nature of this protection was not discussed and it should be understood that conversion of a thiol to a hydropersulfide does represent an oxidation. More recently, it has been proposed that conversion of a protein thiol to a hydropersulfide results in a species that when oxidized further is easily reversible (since oxidation of the sacrificial sulfane sulfur would be expected) under cellular reducing conditions 25, 50. Thus, irreversible oxidative modifications of protein hydropersulfides would not occur as they would for thiol functions in proteins. Importantly, selenoproteins function similarly in that they are also resistant to overoxidation to irreversibly modified species 38, 51.

Clearly, other aspects of hydropersulfide chemistry may be important to their biological utility. Although not unequivocally demonstrated, the formation of a hydropersulfide at a protein active site cysteine could confer increased nucleophilic reactivity, possibly increasing enzyme activity. Thus, thiol proteins that may have important function in an oxidative and/or electrophilic stress environment may have increased activity if converted to a hydropersulfide. Also, as alluded to previously, the more physiologically accessible redox couple for RSS^−^/RSS· compared to RS^−^/RS· may allow the oxidation of a protein cysteine to the corresponding hydropersulfide to serve as an electron transfer “redox gate” 41. That is, electron transfer through an RS^−^/RS· couple is prohibitive since RS· is too oxidizing and RS^−^ is too poor a reductant – however, oxidation of the protein thiol to a hydropersulfide could allow electrons to flow since RSS^−^ is a reasonably good reductant and RSS· is not nearly as oxidizing as RS·. Moreover, RSS· has been shown to be relatively unreactive toward the paramagnetic signaling species NO and O_2_, allowing the RSS^−^/RSS· couple to be usable in aerobic and NO‐rich environments 41, 44. It should be stressed that this idea is speculative at this time and there are no documented examples of this phenomenon. This is presented only because the chemical properties would predict such a scenario.

Clearly, there is a great deal of work to be done to fully elucidate hydropersulfide physiology and the associated mechanisms. The recent work of the Akaike group demonstrating translational incorporation of cysteine hydropersulfides into proteins reveals the importance of hydropersulfides in fundamental biochemistry and provides ample impetus for the further elucidation of hydropersulfide chemistry and biology. Some of the more pressing questions regarding biological hydropersulfides are the following:

### How is hydropersulfide incorporation into proteins regulated?

Protein hydropersulfides are translationally incorporated *via* the actions of CARS. What regulates this activity (i.e., what are the factors that determine whether CARS incorporates Cys‐SH or Cys‐SSH into proteins)? Also, protein hydropersulfides can conceivably be incorporated into proteins *via* sulfur exchange chemistry (i.e., protein cysteines reacting with hydropersulfides or oxidized protein cysteines reacting with H_2_S). With this in mind, it will be important to determine which process, translational incorporation or post‐translational sulfur exchange chemistry, is the most relevant and is the relevance variable depending on cellular conditions? It will also be important to determine the specific roles (direct or indirect) of the transsulfuration enzymes such as 3‐mercaptopyruvate sulfurtransferase (3MST), which is another protein capable of synthesizing hydropersulfides, in hydropersulfide production.

The temporal aspects of hydropersulfides in proteins represent an important issue in delineating the signaling and function of this modification. Thus, it is essential to determine how and when hydropersulfides are generated as well as determine how and when they are removed. In this regard, Doka and coworkers 28 have reported that intracellular protein hydropersulfide levels are highly dependent on the presence and activity of thioredoxin (Trx), an important enzyme involved in the reduction of oxidized thiol proteins. Thus, it will be important to consider the expression, activity, and regulation of Trx (and possibly other reductases) when speculating on the function and signaling associated with protein hydropersulfides.

### What determines which protein cysteines end up as hydropersulfides?

It appears that only certain cysteine residues in proteins are “sulfurated” (33 for a discussion of this term) during translation, and possibly during sulfur exchange processes. Since amino acid incorporation into proteins is governed by codons, it will be important to discern how CARS interprets codons as a mechanism for specific hydropersulfide incorporation into proteins. With respect to post‐translational sulfuration, it is appropriate to mention studies of the enzyme rhodanese, a protein with a catalytic cysteine that is known to be sulfurated. Rhodanese is capable of transferring a sulfane sulfur from a donor molecule to cyanide ion (^−^CN) to make thiocyanate (^−^SCN) *via* an intermediary catalytic cysteine hydropersulfide. Numerous proteins have been identified to possess a “rhodanese homology domain” associated with their catalytic cysteine residues, implicating these residues as potential sites of sulfuration 14, 18, 52. For example, the phosphatase Cdc25, involved in cell‐cycle regulation, possesses a catalytic rhodanese homology domain 53, possibly implicating persulfide formation as a regulatory mechanism in this system.

### What cellular conditions are conducive to hydropersulfide formation?

It has been hypothesized that oxidative and/or electrophilic stress can lead to increases in hydropersulfide generation. This idea is largely based on the fact that hydropersulfides are superior to thiols as reductants and nucleophiles; chemical attributes that may be important in combating oxidative and electrophilic stressors. In partial support of this idea, it has been shown that increased expression of CSE in A549 cells (a lung epithelial cell line) led to increased intracellular hydropersulfides and increased resistance to H_2_O_2_ toxicity 27. Although this result is consistent with the idea that hydropersulfides are protective against oxidative/electrophilic stress, clearly further work must be performed to fully address this hypothesis.

### Why are there multiple biosynthetic pathways for hydropersulfides?

The recent discovery of CARS‐mediated Cys‐SSH generation begs the question of why there are multiple and biochemically distinct processes capable of making the same molecules (i.e., CSE/CBS, CARS, etc.). Clearly, some rationale can include compartmentalization of Cys‐SSH for disparate biochemical purposes, differential regulation for accommodating varying cellular stresses/signals or simple redundancy to ensure the availability of a crucial biological effector. Answering this fundamental question will greatly assist in determining the physiological significance of hydropersulfides. Regardless, the fact that there are multiple processes to generate Cys‐SSH is consistent with the idea that hydropersulfides are important biological species with varied and essential functions.

### How does hydropersulfide incorporation into a protein affect protein activity/function?

As mentioned above, numerous proteins have been reported to possess hydropersulfide residues, at least part of the time. However, it is not clear how hydropersulfides affect the activity and function of all of these proteins. Based on the current literature, it appears that hydropersulfide formation mostly confers a loss or decrease in enzyme activity. For example, the cysteine‐hydrolase papain 22 and the cysteine dehydrogenase GAPDH 26 are inhibited when sulfurated. Sulfuration of erythrocyte enzymes *via* exposure to a hydropersulfide generating system led to the inhibition of numerous enzymes 54. Similarly, liver enzymes treated with CSE/cystine (a system that generates Cys‐SSH) were also found to be either inhibited or unaffected 55. However, hydropersulfide formation in the protein parkin has been reported to enhance its catalytic activity, possibly leading to a protection against the development of Parkinson's disease 56. Further studies are clearly warranted to investigate the possible alteration of protein activity as well as determination of possible functional changes.

### What are the chemical properties that are important to the biological utility of hydropersulfides?

As discussed previously, hydropersulfides should possess all of the chemical properties of a thiol, albeit enhanced. Although this is clear, it is not known which, if any, of these chemical properties are important to hydropersulfide function in biological systems. The enhanced chemistry may not yet be realized at the active sites of proteins. It should also be noted that the chemistry of a hydropersulfide function is, to a certain extent, partly disulfide and partly selenol. Since selenols and disulfides are already established biological effectors, it will be important to determine how and why the functions of hydropersulfides are distinct from the functions of these species. Also, the one‐electron redox chemistry of hydropersulfides and thiols are distinct, with RSS^−^/RSSH being superior reductants compared to RS^−^/RSH and RSS· being much poorer oxidants compared to RS·. Nature has found a use for RS· as an oxidant in numerous enzymatic systems 6. Since RS· is a much stronger oxidant than RSS·, the use of RSS· as an oxidant in analogous systems is not expected. The question remains, however, whether Nature has also found a use for RSS· or the RSS^−^/RSS· redox couple. The stability of RSS· indicates that RSS^−^/RSSH can conceivably act as a one‐electron reductant similar to the antioxidants ascorbate and tocopherols. Indeed, the antioxidant properties of RSSH have already been described 42 and it is intriguing to speculate that endogenously generated hydropersulfides also function in this manner. Interestingly, numerous enzymes proposed to possess hydropersulfides also have redox activity. For example, xanthine oxidase, an enzyme that catalyzes the oxidation of (hypo)xanthine with subsequent reduction of O_2_ to superoxide ( $O_{2}^{-}$ ), was among the first to be proposed to contain a hydropersulfide 10. Regardless, it remains to be determined whether the RSS^−^/RSS· couple has biological utility.

### What are the biological roles and functions of the higher order polysulfide species?

Although this article has focused mostly on RSSH species, it has been mentioned that polysulfides (RSS_n_R, *n* > 1, R = hydrogen or alkyl) are also prevalent and related to hydropersulfides. Indeed, the generation of these higher order polysulfides is a result of H_2_S and hydropersulfide formation since all of these species can be related *via* a series of equilibria. In fact, it is going to be difficult to distinguish among the possible biological activities of all these species since they are related to each other *via* equilibrium reactions and, therefore, can all be present simultaneously in a biological milieu. Regardless, it is entirely possible, if not probable, that polysulfides are not merely storage forms of H_2_S or RSSH species, but rather possess important biological activities and properties on their own. Discerning these activities/properties will be important in the full elucidation of the chemical biology and physiology of the hydropersulfides.

### Are hydropersulfides involved in metal/metalloprotein biology?

Thus far, much of the focus on hydropersulfide biochemistry has primarily involved interactions with other sulfur species (*vide supra*). Due to the presumed novel ligation and redox properties of hydropersulfides, metals and metalloproteins are also likely sites of biological reactivity. Previous studies have demonstrated that the RSSH species can reduce ferric heme proteins such as cytochrome c under conditions where thiols or H_2_S do not 22 (and, as mentioned above, the biological relevance/utility of the one‐electron redox properties of hydropersulfides are worthy of further investigation). It also seems possible, if not likely, that hydropersulfides will interact with biologically nonredox active metal ions such as Zn^2+^ as well. As a borderline “soft” ion, Zn^2+^ can coordinate a wide array of biological ligands containing S, N, and O atoms. For example, Zn‐finger proteins bind Zn^2+^ *via* histidine nitrogens and cysteine thiols 57. The regulation of Zn^2+^ “redistribution” (i.e., movement of Zn^2+^ from storage sites to protein binding sites) is an important field due to the prevalence and of Zn‐containing proteins in signaling fundamental biochemistry 58. Indeed, one mechanism of mobilization of Zn^2+^ from sites of sequestration is thought to be *via* redox chemistry at Zn‐binding thiolates, coupling redox signaling with Zn‐dependent signaling/biochemistry. It is intriguing to speculate that cysteine persulfide anions can also be involved in Zn^2+^ coordination, allowing a different redox couple (compared to thiolate) to affect Zn^2+^ binding, release and/or mobilization. Moreover, it is highly likely that the coordination chemistry of a persulfide anion versus a thiolate will be significantly different, thus altering the affinity of proteins for Zn^2+^ and possibly resulting in a chemically distinct pool of Zn^2+^ (e.g., where the redox chemistry of the Zn^2+^ ligands allows for release under different redox conditions).

One aspect of the interaction of hydropersulfides with metals in a biological system that has been examined is the effect of persulfides on electrophilic heavy metal toxicity 59. Abiko and coworkers 60 found that detoxification of methyl mercury can occur *via* interaction with H_2_S, hydropersulfides and polysulfides. Similarly, Cd^2+^ toxicity can also be abated by the presence of hydropersulfides 61. Importantly, Zn^2+^ and Cd^2+^ are chemically and biochemically similar in that they both readily bind thiol proteins and indeed a facet of Cd^2+^ toxicity is thought to involve interference with Zn^2+^ homeostasis 62. The fact that Cd^2+^ and Zn^2+^ are chemically similar and persulfides alter the pathobiology of Cd^2+^ is consistent with the idea that endogenous persulfides can also be involved in Zn^2+^ regulation.

### Are hydropersulfides involved in the etiology of disease or involved in redox signaling?

One of the most important aspects of hydropersulfide/polysulfide chemical biology is with respect to their possible roles in disease. As alluded to above, hydropersulfides possess the proper chemical properties to protect cells from certain chemical stresses, although this remains to be established. Moreover, hydropersulfides possess chemical properties that are distinct from thiols (and other derived species), allowing them to perform biochemistry not available to simple thiols. Considering all of this, these species may be involved in many diseases, disorders or developmental pathways whereby “oxidative” or electrophilic stress and/or signaling have been implicated and/or where thiol modification processes are proposed. Moreover, if hydropersulfides are fundamental to cellular protection, they may also be important aspects of, for example, cancer cell viability, neuronal cell degeneration, regulation of apoptosis, and/or redox‐mediated developmental pathways. These are highly speculative ideas, but it is clear that the impact of hydropersulfides on disease states, disorders, developmental signaling, or cellular protection is potentially significant.

The points listed above likely represent only the “tip‐of‐the‐iceberg” with regard to pressing questions in this field. Moreover, details of the physiological implications of hydropersulfides and polysulfides remain to be addressed once some of the above questions are answered. It appears likely that the current ideas regarding the biological signaling associated with thiol sulfur modification will need to be revisited and redefined in the light of the recent findings pertaining to the prevalence of hydropersulfides and polysulfides. Moreover, hydropersulfide/polysulfide‐derived species that are analogous to established thiol‐derived species will also be essential to include in any conversation involving sulfur species. For example, thiol oxidation to the corresponding sulfenic acid is a well‐known and established process under certain biological conditions 63. It will now be equally important to consider the analogous reaction with hydropersulfides under those same conditions. That is, oxidation of a hydropersulfide to the corresponding perthiosulfenic acid (RSSOH) is an equally likely, if not more likely, fate for hydropersulfides 64. It is unnecessary to list all of the biologically relevant thiol modification reactions but yet important to indicate that any type of thiol modification reaction (many of which have been associated with thiol‐based signaling) can occur and are likely with hydropersulfides, possibly with important signaling/biochemical consequences. Regardless, the potential importance and impact of hydropersulfides and polysulfides on the fields of fundamental biochemistry and (patho)physiology, among many others, appear significant and burgeoning and, in our opinion, it would be unwise not to consider these novel species as important molecules in the chemical biology of nature.

## Note

1 The term “sulfhydration” was used to denote the addition/insertion of a sulfane sulfur (basically atomic sulfur) to/into a molecule. That is, the conversion of RSH to RSSH was referred to as “sulfhydration” of RSH. This term may not be the most accurate description of the chemistry since “hydration” describes reactions that are simply represented by the addition of water. For example, the addition of water to an olefin to generate the corresponding alcohol is a hydration reaction. The term “sulfhydration” may have been derived from the term “sulfhydryl”, which has been used to describe an ‐SH group (note that “sulf” denotes a sulfur atom and “hydryl” denotes a hydrogen and is distinct from “hydration”). Thus, it may be tempting to invoke the term “sulfhydrylation”. However, this terminology is also not an accurate description of the chemistry since conversion of RSH to RSSH is not the addition of an ‐SH (sulfhydryl) group but, as described above, the addition of a sulfur atom. Therefore, as Toohey discusses and recommends 33, “sulfhydration” should be replaced by “sulfuration” for these reactions since the conversion of RSH to RSSH is equivalent to the addition of a sulfur atom.
